# Transforming growth factor β: A potential biomarker and therapeutic target of ventricular remodeling

**DOI:** 10.18632/oncotarget.17255

**Published:** 2017-04-19

**Authors:** Yuan Ma, Hai Zou, Xing-Xing Zhu, Jie Pang, Qiang Xu, Qin-Yang Jin, Ya-Hui Ding, Bing Zhou, Dong-Sheng Huang

**Affiliations:** ^1^ Department of Cardiology, Zhejiang Provincial People's Hospital, Hangzhou, China; ^2^ Department of Nephrology, Zhejiang Provincial People's Hospital, Hangzhou, China; ^3^ Department of Cardiac Surgery, Zhejiang Provincial People's Hospital, Hangzhou, China; ^4^ Department of Hepatobiliary Surgery, Zhejiang Provincial People's Hospital, Hangzhou, China; ^5^ People's Hospital of Hangzhou Medical College, Hangzhou, China

**Keywords:** transforming growth factor β, myocardial fibrosis, ventricular remodeling, mechanism, targets

## Abstract

Transforming growth factor β (TGF-β) is a multifunctional cytokine that is synthesized by many types of cells and regulates the cell cycle. Increasing evidence has led to TGF-β receiving increased and deserved attention in recent years because it may play a potentially novel and critical role in the development and progression of myocardial fibrosis and the subsequent progress of ventricular remodeling (VR). Numerous studies have highlighted a crucial role of TGF-β in VR and suggest potential therapeutic targets of the TGF-β signaling pathways for VR. Changes in TGF-β activity may elicit anti-VR activity and may serve as a novel therapeutic target for VR therapy. This review we discusses the smad-dependent signaling pathway, such as TGF-β/Smads, TGF-β/Sirtuins, TGF-β/BMP, TGF-β/miRNAs, TGF-β/MAPK, and Smad-independent signaling pathway of TGF-β, such as TGF-β/PI3K/Akt, TGF-β/Rho/ROCK,TGF-β/Wnt/β-catenin in the cardiac fibrosis and subsequent progression of VR. Furthermore, agonists and antagonists of TGF-β as potential therapeutic targets in VR are also described.

## INTRODUCTION

**Figure 1:**
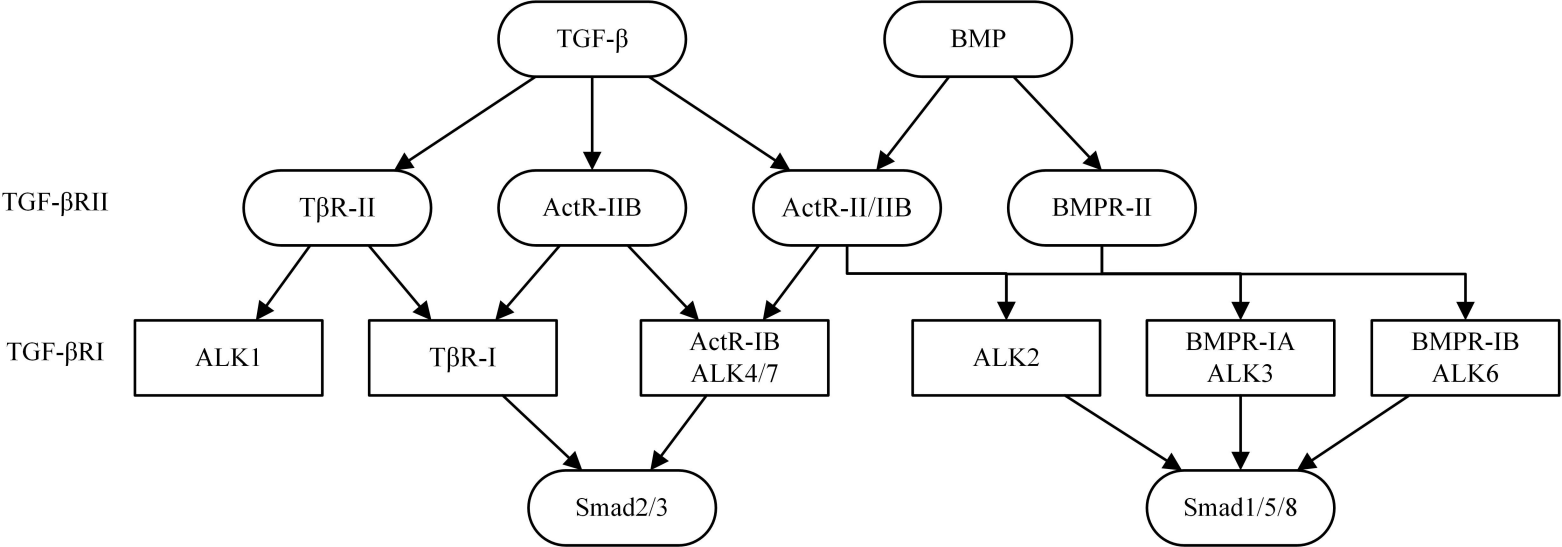
Important ligands of TGF-β signaling pathways in cardiac

Ventricular remodeling (VR) is a complicated process involving cardiomyocyte hypertrophy, inflammation, fibrosis and occurs in response to changes in mechanical and neurohormonal stimulation [1]. VR is characterized by progressive ventricular dilatation, myocardial hypertrophy, fibrosis, and deterioration of cardiac performance, and arises from interactions between adaptive modifications of cardiomyocytes and negative aspects of adaptation such as cardiomyocyte death and fibrosis. VR is defined as structural changes in the left ventricle with three major patterns: concentric remodeling, eccentric hypertrophy, and myocardial infarction [2]. Transforming growth factor β (TGF-β) primarily signals through TGF-β type I receptor (TβRI), also named activin receptor-like kinase (ALK), TβRII and TβRIII. TβRI and TβRII have intrinsic serine/threonine kinase activity and mediate the downstream effects of TGF-β. Recent studies have demonstrated that TGF-β plays a critical role in the regulation of cell growth, differentiation and immune function. In cardiac, TGF-β binds to a complex of type II-R and type I-R (=ALK5), and activin or myostatin, which bind to ALK4, 5, or 7 and active Smad 2 and 3. BMP, which binds to BMPR-II, and ALK2, 3, or 6 and activates Smad 1, 5, or 8 [3] (Figure 1). Sustained pressure overload induces cardiac myocyte hypertrophy and dysfunction along with interstitial changes such as fibrosis and reduced capillary density which are facilitated by TGF-β. The final step in the process of heart failure after pressure overload and myocardial infarction (MI) is cardiac fibrosis which is regulated by TGF-β [1]. TGF-β have received increased and deserved attention in recent years because it may play a potentially novel and critical role in the development and progression of myocardial fibrosis and the subsequent progression of VR. The activation of TGF-β promotes myofibroblast differentiation and transformation, enhances the expression of extracellular matrix (ECM) which participates in a collagen-based scar formation; and inhibits the expression of matrix metalloproteinases (MMPs), which specifically restrains ECM and decrease VR. In this review, we focus on the most extensively investigated TGF-β in VR, and we discuss representative TGF-β signaling pathways and their respective effects on VR. This review we discusses the smad-dependent signaling pathway, such as TGF-β/Smads, TGF-β/Sirtuins, TGF-β/BMP, TGF-β/miRNAs, TGF-β/MAPK, and Smad-independent signaling pathway of TGF-β, such as TGF-β/PI3K/Akt, TGF-β/Rho/ROCK,TGF-β/Wnt/β-catenin in the cardiac fibrosis and subsequent progression of VR. (Figure 1).

### Smad-dependent signaling pathway of TGF-β

#### TGF-β/Smads

Nuclear accumulation of active Smad complexes is crucial for the transduction of TGF-β-superfamily signals from transmembrane receptors into the nucleus. There are nine different Smads that have been identified in mammals, and these Smads can be classified into three subclasses, receptor-activated Smads (R-Smads) (Smad1, 2, 3, 5, 8 and 9), inhibitory Smads (I-Smads) (Smad6 and Smad7), and common-partner Smads (Co-Smads) (Smad4) [4]. Stimulating receptors can phosphorylate R-Smads, which forms oligomeric complexes with Co-Smads. I-Smads can suppress the signals from the serine/threonine kinase receptors [5]. Accumulating evidence has shown that cardiac remodeling is regulated by the TGF-β/Smad signaling pathway.

#### R-Smads

The effect of Smad2 and Smad3 on R-Smads has been most widely studied in the process of myocardial fibrosis in recent years. A study reported that high glucose levels enhanced p300 activity, which increased TGF-β activity via Smad2 acetylation, thus promoting cardiac fibrosis, cardiac hypertrophy and diastolic function impairment [6]. Another study showed shown that angiotensin II (AngII) induced left ventricular fibrosis and remodeling, which were dependent on both Smad2 and extracellular regulated protein kinase (ERK) activation, and could be inhibited by the AT1 receptor [7]. Smad3 exerts a similar effect to Smad 2 in regulating cardiac fibrosis. One study suggested that activation of Smad3 was important in fibrotic response and cardiac fibroblast (CF) activation post-MI [8]. These results are consistent with a study that Smad3 deficiency attenuated bleomycin-induced pulmonary fibrosis in mice [9]. It is noteworthy that, many studies have found the inhibitor of Smad2 or Smad3 to found an important role in the progression of ventricular fibrosis and VR. Chen et al. showed that beraprost, which is a prostacyclin analog that can significantly block TGF-β expression and Smad2 phosphorylation, suppressed the proliferation of CFs [10]. Another study suggested that glycogen synthase kinase 3β (GSK-3β), a small-molecule inhibitor of Smad3, largely suppressed fibrosis and limited left VR [11]. Another study also showed that through abrogating the phosphorylation of Smad2 and Smad2/3 nuclear translocation, taxifolin remarkably inhibited left ventricular fibrosis and collagen synthesis [12]. Moreover, many other inhibitors of Smad2 or Smad3, such as AVE 3085 [13] and growth/differentiation factor 1 [14], have the ability to suppress VR.

#### I-Smads

Smad7, one of the I-Smads, has been shown to inhibit fibrosis and inflammation in many kidney diseases, however, study has shown that decreased Smad7 expression contributed to cardiac fibrosis in the pathogenesis of cardiac fibrosis in the post-MI heart [15]. Recently, TGF-β/Smad7 has been demonstrated to be important not only in kidney diseases, but also in cardiac diseases. In an analysis of AngII-induced VR, Wei et al. found that Smad7 attenuated cardiac inflammation and fibrosis, such as by down-regulating IL-1β and TNF-α, inhibited collagen I and α-SMA and suppressed Ang II-mediated VR[16]. A recent study examined the role of Smad7 in spontaneously hypertensive rats (SHRs). It was found that fluvastatin decreased cardiac fibrosis through regulation of TGF-β1/Smad7 [17]. A study of high-mobility group box 1 (HMGB1) which has been reported to decrease VR in the post-MI failing myocardium also supported this result. In rats that the underwent coronary artery ligation, after four weeks of treatment with HMGB1, TGF-β1 and phosphor-Smad2 (p-Smad2) were inhibited, but, Smad7 was increased. In addition, in CFs, HMGB1 enhanced the expression of Smad7 and attenuated the level of collagen I [18]. A study of Smad6, another I-Smad, showed that activation of Smurf1-dependent Smad6 suppressed TGF-β1-induced expression of Smad3 and PKC-δ and collagen deposition [19].

Although the TGF-β/Smads signaling pathway has been demonstrated to inhibit VR, some evidence has shown that it also promotes VR. (Table 1) More research is needed to further elucidate the functional mechanisms of TGF-β/Smads in VR and explore the biology of TGF-β/Smads for their potential use in the clinical treatment of VR.

**Table 1:** Smad signaling pathway of TGF-β

Regulatory factor	Smad	Effect for target	Effect for TGF-β	Effect for ventricular remodeling	Reference
AngII	Smad2, ERK	active	active	induced LV fibrosis and remodeling	[7]
p300	Smad2	active	active	promoted cardiac fibrosis	[6]
Androgens	Smad2	active	active	Promoted myocardial remodeling	[65, 66]
MSC	Smad2	active	active	promoted myofibroblasts congregating	[67]
eNOS/NOS	Smad2	negative	negative	improved ventricular remodeling after myocardial infarction	[68]
beraprost	Smad2	negative	negative	suppressed proliferation of cardiac fibroblast	[10]
SM16	Smad2	negative	negative	Attenuated myocardial remodeling	[69]
Caveolin-1	Smad2	negative	negative	Attenuated cardiac remodeling	[70]
bgn	Smad2	negative	negative	Attenuated extracellular matrix remodeling	[71]
MG132	Smad2	negative	negative	attenuated cardiac remodeling	[72]
leptin	Smad2	negative	negative	prevented cardiac fibroblast activation and collagen production	[73]
atorvastatin	Smad2	negative	negative	improved cardiac remodeling	[74]
GW788388	Smad2	negative	negative	attenuated left ventricular remodeling	[75]
BNP	Smad2	negative	negative	prevented ventricular remodeling	[76]
GSK-3β	Smad3	negative	negative	suppressed cardiac fibrosis and limited left ventricular remodeling	[11]
TAX	Smad2,3	negative	negative	inhibited left ventricular fibrosis and collagen synthesis	[12]
Paeoniflorin	Smad2,3	negative	negative	inhibited cardiac remodeling	[77]
Tranilast	Smad2,3	negative	negative	reduced pathological fibrosis following myocardial infarction	[78]
AVE3085	Smad2,3	negative	negative	Attenuated cardiac remodeling	[13]
PNFE	Smad2,3	negative	negative	Improved left ventricular remodeling	[79]
SBTI	Smad2,3	negative	negative	Improved left ventricular remodeling	[79]
HCTZ	Smad2,3	negative	negative	improved cardiac remodeling	[80]
GDF1	Smad2,3, ERK1/2	negative	negative	attenuated cardiac remodeling	[14]
H2S	Smad2,3	negative	negative	prevented myocardial remodeling	[81]
BMP2	Smad6	active	negative	Improved cardiac fibrotic	[19]
fluvastatin	Smad7	active	negative	decreased cardiac fibrosis	[17]
HMGB1	Smad7	active	negative	decreased ventricular remodeling	[19]
SBTI	Smad7	active	negative	Improved left ventricular remodeling	[79]
PNFE	Smad7	active	negative	Improved left ventricular remodeling	[79]
intermedin 1-53	smad3	negative	negative	decreased cardiac fibrosis	[82]
Osthole	Smad2,3	negative	negative	decreased cardiac fibrosis	[83]
Osthole	Smad7	active	negative	decreased cardiac fibrosis	[83]
SP	Smad2,3	negative	negative	decreased cardiac fibrosis	[84, 85]
CB2 receptor	Smad3	negative	negative	decreased cardiac fibrosis	[86]

AngII = angiotensin II; MSC = mesenchymal stem cells; eNOS = endothelial nitric-oxide synthase; NOS = nitric oxide system; SM16 = small molecule inhibitor 16; bgn = biglycan; BNP = B-type natriuretic peptide; GSK-3β = glycogen synthase kinase-3; TAX = taxifolin; PNFE = panax notoginseng flower extract; SBTI = soybean trypsin inhibitor; HCTZ = hydrochlorothiazide; GDF1 = growth/differentiation factor 1; H2S = hydrogen sulfide; BMP = bone morphogenetic protein; HMGB = high-mobility group box; SP = substance P.

#### TGF-β/Sirtuins

Sirtuins are a group of histone deacetylases (HDACs) consisting of Sirt1-Sirt7. Sirtuins regulated the activity of proteins and enzymes, and maintains the stability the enzymes and proteins by the acetylation of lysine residues. Some Sirtuins, such as Sirt1 [20], Sirt3 [21], Sirt7 [22], also play an important role in VR.

#### Sirt1

Sirt1is categorized as a class III HDAC which negatively regulates the expression of Smad7 and thereby promotes TGF-β/Smad-dependent transcription. Moreover, Sirt1 attenuates the expression of peroxisome proliferator-activated receptor, which is an important inhibitor of TGF-β signaling. One study examined the role of Sirt1 in regulating TGF-β/Smad signaling in systemic sclerosis. The results showed that knockdown of Sirt1 could effectively suppress TGF-β signaling and exert anti-fibrosis effects [23]. VEGF has been demonstrated to attenuate hypertensive left VR, which was induced by high salt intake [24]. Another study showed that TGF-β-stimulated VEGF was attenuated by resveratrol, at least in part, by Sirt1 activation [25].

#### Sirt3

Sirt3 has been shown to be related to longevity in humans [26]. However the molecular mechanistim of this longevity is still in disputed, although the protective effect of Sirt3 on cardiomyocytes has been demonstrated. A recent study shoed that over-expression of Sirt3 protected cardiomyocytes against genotoxic and oxidative stress [27]. Another study showed that Sirt3, induced by resveratrol, suppressed the transformation of fibroblasts-to-myoblasts through the TGF-β/Smad3 pathway in response to AngII in isolated CFs [21].

#### Sirt7

Sirt7 is primarily localized in the nucleoli and regulates RNA polymerase I transcription. It is well known to play a critical role in human carcinoma and lipid metabolism. Apart from these roles, it has also been reported that Sirt7 contributes to myocardial tissue repair. Araki et al. showed that the autophagy inhibitor attenuated TβRI down-regulation, which was induced by the absence of Sirt7 [22]. Moreover, the loss of Sirt7 activated autophagy in cardiac fibroblasts. The data showed that Sirt7 maintains TβRI by modulating autophagy and plays an important role in suppressing rat CFs and increasing myocardial tissue repair [22]. Sirt7 seems a promising therapeutic target for VR. These studies suggest that Sirtuins have an important role in the procession of VR through the TGF-β pathway and this role may be utilized in the development of a series combination therapies that target Sirtuins in patients with VR (Table 2)

**Table 2:** Sirtuins signaling pathways of TGF-β

Regulatory factor	Effect for Sirt	Effect for Smad	Effect for TGF-β	Effect on ventricular remodeling	Reference
	Sirt1	↓Smad7	active	promoted ventricular remodeling	[23–25]
resveratrol	↑Sirt3	↓Smad3	negative	prevented cardiac fibrosis	[21]
	Sirt7		negative	prevented cardiac fibrosis	[22]

TGF-β = transforming growth factor β.

#### TGF-β/ BMPs

BMPs play a critical roles in cardiac progenitor specification, proliferation and differentiation [28]. Additionally, BMPs can attenuate adverse fibrosis progression [29]. It has been reported that in renal interstitial fibroblast cells, over-expression of BMP-2 suppressed fibrosis, induced by TGF-β1 by increasing the catabolism of TGF-βRI [19]. One study showed that *in vitro* cultured cardiomyocytes and BMP-2 suppressed TGF-β1 through the activation of Smurf1/Smad6 complex. Moreover, in the mouse heart, after 14 days of treatment with rhBMP-2, overload-induced collagen deposition by pressure was decreased, and TGF-β1-dependent activation of Smad3 and PKC-δ was attenuated (Table 3) [19].

**Table 3:** BMPs signaling pathways of TGF-β

Regulatory factor	Effect for BMP	Effect for Smad	Effect for TGF-β	Effect on ventricular remodeling	Reference
	BMP2	↑Smad6	negative	improved cardiac fibrotic	[19]

TGF-β=transforming growth factor β; BMP=bone morphogenetic protein.

#### TGF-β/ miRNAs

It has been reported that, in the heart, some microRNAs (miRNAs), such as miR-29, miR-133, and miR-30 regulate the expression of ECM proteins and collagens [30, 31]. In recent studies, other miRNAs have been demonstrated to regulate cardiac fibrosis through the TGF-β signaling pathway. Nagalingam et al. suggested that miR-378 deficiency to the development of cardiac fibrosis through a TGF-β-dependent mechanism, in cardiomyocytes [32]. Villar et al also found miR-21 to be a biomarker for myocardial fibrosis in aortic stenosis patients [33]. Rana et al. found a similar result, in the MI heart, miR-21 and miR-29b contributed to cardiac fibrosis via a mechanism involving the TGF-β1 signaling pathway [34]. Zhao et al. identified that in CFs, miR-101a suppressed cardiac fibrosis, which was induced by hypoxia through the TGF-β signaling pathway [35]. In the study of cardiac hypertrophy and fibrosis by Tijsen et al. The miR-15 family was found to suppress hypertrophy and fibrosis by inhibiting the TGF-β pathway [36]. Many other miRNAs have been demonstrated to play an important role in the TGF-β pathway associated with myocardial fibrosis, such as miR-24 [37], miR-26 [38] miR-31 [39], miR-34a [40], miR-122 [41], and miR-208a [42]. There is some evidence to indicating that the activation or inhibition of specific TGF-β/miRNAs may be beneficial for VR patients and raising the possibility that TGF-β/miRNAs could be a therapeutic target for drug discovery.

#### TGF-β/MAPK

The mitogen-activated protein kinase (MAPK) signaling pathway has three kinases: MAP kinase kinase kinase (MKKK), MAP kinase kinase (MKK) and MAPK. MAPK has four subtypes, ERK1/2, c-Jun NH 2-terminal kinase (JNK), p38MAPK and ERK5.

#### ERK1/2

A study suggested that, in lung fibrosis, ERK1/2 signaling played an important role in protease-activated receptor 1 (PAR1)-mediated pro-fibrotic activity [43]. Furthermore, TGF/ERK1/2 also exerted an important role in cardiac tissue. One study showed that, SCH79797, which is an inhibitor of PAR1, blunted ERK1/2 phosphorylation, TGF-β and type I pro-collagen production and myofibroblasts transformation in isolated CFs [44]. Li L et al. found that, in cultured adult rat CFs, ERK1/2 took part in periostin, which is a key regulator of cardiac fibrosis, expression through TGF-β1 pathway regulation [45]. In an analysis of farnesyltransferase inhibition, Li et al. found that farnesyltransferase inhibition attenuated myocardial fibrosis and improved VR in SHRs partly through suppression of the ERK1/2 phosphorylation pathway [46].

#### JNK and p38 MAPK

One study revealed that tissue kallikrein attenuated left VR, improved cardiac function and prevented inflammation after myocardial ischemia/reperfusion (I/R) through kinin B2 receptor activation and NO formation partly through the suppression of the JNK/p38 MAPK signaling pathway [47]. However, another study showed that in SHRs, oxymatrine (OMT) attenuated VR by inhibiting the over-expression of angiotensin converting enzyme (ACE) and TGF-β1, thereby attenuating ERK 1/2, JNK and p38 MAPK signaling pathway activation [48]. Similar results were also found in a study of streptozotocin (STZ) induced diabetes in mice. Diabetic mice were treated with alpha-lipoic acid (ALA), resulting in the mitigation of JNK and p38 MAPK activation and attenuation of interstitial fibrosis [49]. Matsumoto-Ida et al. also suggested that, in rats, the TGF-β1-TAK1-p38 MAPK signaling pathway played a vital role in left VR after MI [50]. A further study made by Sriramula et al. showed that TNF-α contributed to angiotensin II induced hypertension and adverse VR the through MAPK(JNK and p38 MAPK) /TGF-β/NF-κB pathway [51]. (Table 4) In conclusion, TGF-β/ MAPK modulation could potentially be a novel therapeutic approach for the prevention and treatment of VR.

**Table 4:** MAPK and PI3K/Akt signaling pathways of TGF-β

Regulatory factor	Expression levels in ventricular aneurysm	Antagonist	Agonist	Effect on ventricular remodeling
ERK1/2	up-regulated	SCH79797 [44] oxymatrine(OMT) [48]		active
JNK/p38 MAPK	up-regulated	Kallikrein [47]	TNF-α [51]	active
PI3K/Akt	up-regulated	Atorvastatin [54]		

TGF-β = transforming growth factor β; ERK = extracellular regulated protein kinases; JNK = c-Jun NH 2-terminal kinase; MAPK = mitogen activated protein kinase; PI3K/Akt = phosphatidylinositol-3 kinase/protein kinase B; GDF1 = growth/differentiation factor 1; cAMP = cyclic adenosine monophosphate; OMT = oxymatrine.

#### Other smad-dependent signaling pathway

It was worth noting that, some other new smad-dependent signaling pathways were discovered in recent years. Such as endoglin [52, 53], fibulin-2 [54], serpine1 [55], serpineE2 [56]. Tseliou et al. found that, in rodent models of acute myocardial infarction, cardiospheres (CSps) secreted soluble endoglin and attenuate remodeling by inhibiting TGF-β1/smad signaling [52]. Kapur et al. also found that soluble endoglin limited TGF-β1 signaling in cardiac fibroblasts and attenuated cardiac fibrosis in an *in vivo* model of heart failure [53]. Khan et al. found Ang II cannot induce TGF-β activation without fibulin-2 and that fibulin-2 has an essential role in Ang II-induced TGF-βsignaling and subsequent myocardial fibrosis [54]. Study showed that angiotensin II (Ang II) played a critical role in the cardiac remodeling, however, this effect could be improved by serpine1 in a mouse model [55]. Study showed that serpinE2 significantly were increased with collagen accumulations induced by TGF-β stimulation *in vitro*. And the ERK1/2 signaling promoted the activation of serpinE2, consequently led accumulation of collagen protein, and contributed to cardiac fibrosis [56].

### Smad-independent signaling pathway of TGF-β

#### TGF-β/ PI3K/Akt

It has been reported that TGF-β1 up-regulated phosphatidylinositol-3 kinase/protein kinase B (PI3K/Akt) signaling molecules in human lung fibroblasts, mouse mesangial cells and embryonic fibroblasts [57]. Similar to these studies, Voloshenyuk TG et al. found that, in CFs, TGF-β1 augmented collagen expression and required activation of the PI3K/Akt signaling pathway, suggesting that the PI3K/Akt pathway may be involved in TGF-β1 signaling [58]. Shyu et al. also discovered, in CFs, that PI3K/Akt phosphorylation was up-regulated and that the expression of collagen I was also increased in response to TGF-β1 (Table 4) [59].

#### TGF-β/ Rho/ROCK

Rho-associated protein kinase (ROCK) is a serine/threonine kinase that has been demonstrated to exert a vital role in several cardiovascular diseases, such as coronary vasospasm, hypertension, vascular inflammation and I/R injury [1]. In CFs, study has demonstrated that Rho/ROCK plays a crucial role in mediating several profibrotic responses [60]. Furthermore, it has been demonstrated that TGF-β can signal through Rho/ ROCK pathways [61], and that Rho signaling is vital to the transdifferentiation of myofibroblasts [62]. Li et al. showed that, facial, which is an inhibitor of ROCK, prevented cardiac fibrosis in response to transverse aorta (TAC) and MI. Moreover, this effect of Rho was associated with the up-regulation of profibrotic gene expression and the TGF-β1-TAK1 signaling pathway [1]. Another study revealed that TGF-β1-induced ROCK up-regulation suppressed the expression of BMP-2, which enhanced cardiac fibrosis [19].

#### TGF-β/ Wnt/β-catenin

The Wnt/β-catenin signaling pathway has been reported to be related to pre-natal development, cell division, cell regeneration, stem cell generation and other cellular processes. Cross-talk between the Wnt/β-catenin and TGF-β pathways has been studied. Akhmetshina et al. showed that canonical Wnt signaling was necessary for TGF-β-induced fibrosis [63]. Another study showed that miR-29 mediated TGF-β1-induced ECM synthesis by increasing the pathway of Wnt/β-catenin in human orbital fibroblasts [64] We could predict that in the process of CFs, TGF-could predict the Wnt/catenin signaling pathway and played an important role in the regulation of fibrosis and VR.

## CONCLUSIONS

**Figure 2:**
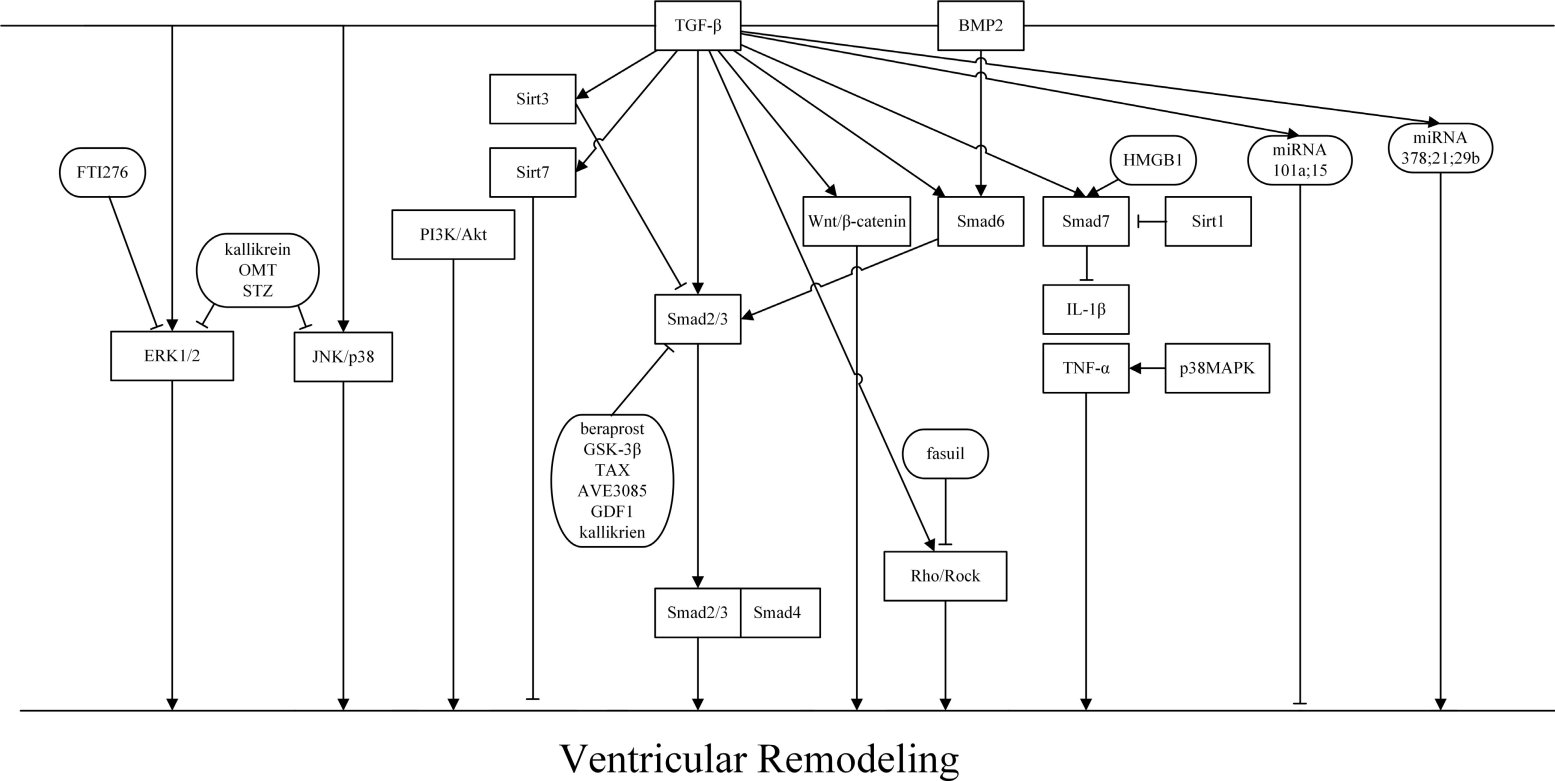
TGF-β signaling pathways and the role of TGF-β in VR TGF-β transduces its signal through Smad-dependent and Smad independent pathways.

TGF-β has been demonstrated to exert biological effects through dependent or Smad-independent signaling pathways. Figure 2 In Smad-dependent signaling pathways, increasing the activation of TGF-β/smad1/5 or TGF-β/smad2/3 resulted in augmenting the expression of CFs. However, activating Smad6/7 could inhibit CFs. Not only did TGF-β/Smads play a dual role in the regulation of TGF-β, but sirtuins also played an important role in regulating TGF-β. Of the sirtuins, Sirt1 had the ability to negatively regulate the expression of Smad7 and decrease the inhibition of TGF-β/Smad7, thereby decreasing fibrosis. However, Sirt3 has been reported to inhibit cardiac fibrosis mainly by inhibiting Smad2/3 and Sirt7 through direct suppression of CFs. As a member of the TGF-β superfamily, BMPs have been reported to play an important role in VR. BMPs can attenuate adverse fibrosis progression. BMP2 was be suppressed by Wnt/β-catenin and promoted Smad6 to suppress cardiac fibrosis by attenuating Smad2/3 with the assistance of Smurf1. In Smad-independent signaling pathways, TGF-β interacted with other signaling pathways to regulate myocardial fibrosis and VR. In the TGF-β/MAPK signaling pathway, TGF interacted with ERK1/2, JNK, and p38 MAPK, playing an active role in myocardial fibrosis.FTI276 could suppress ERK1/2 phosphorylation, and kallikrein, OMT, and STZ could inhibit ERK1/2 and JNK/p38 MAPK phosphorylation to decrease VR. In other Smad-dependent signaling pathways, TGF-β1 mediated the augmention of collagen expression by activation of PI3K/Akt [58]. Fasuil inhibited the activation of Rho/ROCK to prevent cardiac fibrosis in response to TAC and MI. Moreover, Rho is associated with up-regulation of the TGF-β1-TAK1 signaling pathway [1]. miRNAs are currently a relatively popular research topic. However, some miRNA, such as miR-101a, miR-15, and miR-29, inhibit cardiac fibrosis. Other miRNAs could be used as biomarkers for myocardial fibrosis in aortic stenosis patients. Therefore, TGF-β may be a potential therapeutic target for the detection and therapy for VR. Because the biological and molecular mechanisms of TGF-β in ventricular aneurysm are still entirely unknown, it is necessary for further research to help elucidate the signaling pathways involved.

## EXPERT OPINION

In this report, we have discussed the role of TGF-β in VR and the potential use of TGF-β signaling pathways as sources of therapeutic targets for VR based on recent studies. To date, several studies on the mechanisms of action of TGF-β have been conducted, and an increasing number of experts have highlighted the important role of TGF-β signaling pathways in the progression of myocardial fibrosis and subsequent progression of VR. (Figure 1). By investigating one of the most widely studied signaling pathways, namely, TGF-β, we made several interesting observations. The first observation is that Smads dually regulates VR. Some activators, such as Ang II, p300, and arogens, induce VR through activation of Smad 2;however, Some activators, such as BMP2, fluvastatin, and HMGB1, improved VR through activation of Smad7 [6, 7, 19, 65]. The second observation is that VR caused by a variety of diseases (hyperglycemia [6], post-MI heart [15], and spontaneously hypertension [17]) can be regulated by TGF-β/Smad signaling pathways and improve VR. The discovery of TGF-β has led to the identification of new approaches to treat VR. To date, much significant research on the mechanisms of action of TGF-β has been conducted, and an increasing number of experts have highlighted the potential association between TGF-β and VR. Furthermore, TGF-β may offer novel potential as a therapeutic target for VR. However, the biological and pathological effects and molecular mechanisms of the TGF-β signaling pathways in VR remain unresolved, and many more studies of TGF-β are needed to determine the potential modulation of TGF-β signaling pathways for the treatment of VR and other human diseases.
